# Adoption of Covid-19 preventive behaviors in the community: Salvador and Rio de Janeiro

**DOI:** 10.11606/s1518-8787.2026060006406

**Published:** 2026-05-01

**Authors:** Gabriele Amorim, Danielle Souto de Medeiros, Fabiane Soares, Laio Magno, Thaís Regis Aranha Rossi, Thiago Silva Torres, Valdiléa Gonçalves Veloso, Débora Castanheira, Inês Dourado

**Affiliations:** I Universidade Federal da Bahia. Instituto Multidisciplinar de Saúde. Programa de Pós-Graduação em Saúde Coletiva. Vitória da Conquista, BA, Brasil; II Universidade Federal da Bahia. Instituto de Saúde Coletiva. Salvador, BA, Brasil; III Universidade do Estado da Bahia. Departamento de Ciências da Vida. Salvador, BA, Brasil; IV Fundação Oswaldo Cruz. Instituto Gonçalo Moniz. Salvador, BA, Brasil; V Fundação Oswaldo Cruz. Instituto Nacional de Infectologia Evandro Chagas. Rio de Janeiro, RJ, Brasil

**Keywords:** COVID-19, Behavior, Disease Prevention, Pandemics

## Abstract

**OBJECTIVE:**

To describe the prevalence of adoption of Covid-19 preventive behaviors and to evaluate associated factors among users of health units in Salvador and Rio de Janeiro.

**METHODS:**

Cross-sectional study conducted between July 2022 and July 2023. Adoption of preventive behaviors was assessed based on eight behaviors, grouped into the following outcomes: social distancing; respiratory etiquette; mask use; hand hygiene; sociodemographic, housing, structural characteristics, and individual perceptions. All analyses were stratified by study site. Bivariate and multivariate analyses were performed using Poisson regression with robust variance.

**RESULTS:**

A total of 5,476 participants from Salvador and 1,940 from Rio de Janeiro were evaluated. The most prevalent preventive behaviors were respiratory etiquette (82.7 and 84.3%) and hand hygiene (84.9 and 79.1%), respectively. In Salvador, age remained associated with all behaviors assessed. In Rio de Janeiro, adoption of preventive behaviors increased among those who received a Covid-19 vaccine booster. In both cities, individuals aged 40–59 years, ≥ 60 years, and those not employed showed higher adherence to social distancing. Respiratory etiquette was more prevalent among women, individuals with complete high school education, and those who received a Covid-19 booster. Mask use and hand hygiene were associated with female sex, older age, and vaccine booster in both locations. Mask use was also more frequent among those vaccinated against influenza, while hand hygiene was associated with higher education.

**CONCLUSIONS:**

These findings reinforce the importance of public policies that promote maintenance of preventive behaviors and awareness of epidemic prevention, particularly among men, younger individuals, those with lower education, and those who do not receive recommended vaccines or booster doses.

## INTRODUCTION

Covid-19 has emerged as a complex public health challenge, significantly affecting population behavior. Global authorities have implemented public health measures aimed at preventing the collapse of healthcare systems, following strategies traditionally used to control infectious epidemics through both preventive and therapeutic actions^1^. Physical distancing, mask usage, adherence to respiratory etiquette and hygiene practices, reduction of population mobility, and quarantine (defined as restricting movement or isolating individuals for a specified period), constituted the primary interventions recommended to mitigate community transmission of Covid-19, alongside social protection policies^1,2^.

In Brazil, political instability hindered the implementation of a unified approach to addressing the Covid-19 epidemic across different levels of government^3^. The president at the time of the pandemic downplayed the importance of adopting preventive behaviors, which adversely affected public adherence. This so-called "Bolsonaro effect" negatively influenced population behavior, leading to higher mortality rates and reduced compliance with physical distancing, Covid-19 vaccination, and other vaccines recommended by the National Immunization Program^4^.

Preventive measures to control Covid-19 have required the adoption and/or adaptation of health behaviors, the acceptance or rejection of which is influenced by individual factors as well as interpersonal and collective relationships. Perceived susceptibility and severity, along with perceived benefits and barriers, are key constructs guiding decision-making. These factors, shaped by sociodemographic, housing, and structural characteristics, may determine the likelihood of accepting or rejecting health behaviors^5^.

Much of the existing research on behavioral changes induced by Covid-19 has concentrated on the peak of the pandemic, between 2020 and 2021, during which both adaptive and maladaptive behaviors were observed^6-9^. However, little is known about the persistence of these behaviors beyond this period, particularly among populations in socioeconomically vulnerable areas.

This study aimed to describe the prevalence of preventive behaviors against Covid-19 and to assess the factors associated with their adoption among users of health units in Salvador/BA and Rio de Janeiro/RJ.

## METHODS

This cross-sectional study was conducted between July 2022 and July 2023 among users of health units located in areas of greater socioeconomic vulnerability in Salvador, specifically in the Cabula-Beiru health district, and in Rio de Janeiro, in Manguinhos. These regions are characterized by social challenges, including inadequate infrastructure, public safety issues, and limited access to essential public services^10,11^. The present study is a subset of the research project "Expansion of Testing, Isolation, Quarantine, e-Health, and Telemonitoring Strategies in the Fight Against Covid-19 in Brazil," also known as the TQT Covid-19 Study^12^.

The TQT Covid-19 Study was conducted in accordance with the guidelines of the Brazilian Research Ethics Committee (*Comitê de Ética em Pesquisa* – CEP). The study protocol was approved by the CEPs of the World Health Organization (CERC.0128A and CERC.0128B) and by local institutional CEPs (Instituto de Saúde Coletiva/Universidade Federal da Bahia – ISC/UFBASalvador: No. 53844121.4.1001.5030; and in Rio de Janeiro – Instituto Nacional de Infectologia/Fundação Oswaldo Cruz – INI/Fiocruz: No. 53844121.4.3001.5240, Escola Nacional de Saúde Pública Sérgio Arouca/Fundação Oswaldo – ENSP/Fiocruz: No. 53844121.4.3001.5240, and Secretaria Municipal de Saúde/Rio de Janeiro – SMS/RJ: No. 53844121.4.3002.5279). All participants provided informed consent prior to participation.

The study sample was non-probabilistic and selected by convenience, consisting of users of the Brazilian Unified Health System (*Sistema Único de Saúde* – SUS) residing in the study regions. For the purposes of this article, valid data from participants aged ≥ 18 years were analyzed.

In healthcare facilities, rapid Covid-19 tests were offered to all individuals who met the epidemiological criteria. Participants were invited to take the test and participate in the research through various awareness strategies: dissemination on the internet and social media, community radio and podcasts, recruitment by health agents, actions within the School Health Program, as well as mobilization of religious institutions and civil society organizations.

Initially, rapid Covid-19 testing and a socio-behavioral questionnaire were administered at the health facility while participants awaited their test results. The questionnaire comprised seven sections: sociodemographic and housing information; comorbidities; access to and utilization of health services; previous Covid-19 infection and testing; vaccination; behaviors, attitudes, and practices related to Covid-19 prevention and self-medication; perception of infection risk and disease severity; and self-testing.

Data collection in Salvador was conducted between July 2022 and April 2023, while in Rio de Janeiro it took place between November 2022 and July 2023. The research team, consisting of health professionals and community agents, received prior training.

To evaluate the factors associated with Covid-19 preventive behaviors, a conceptual model was developed, including sociodemographic, housing, and structural variables, as well as additional variables related to individual perceptions (Figure).

**Figure:**
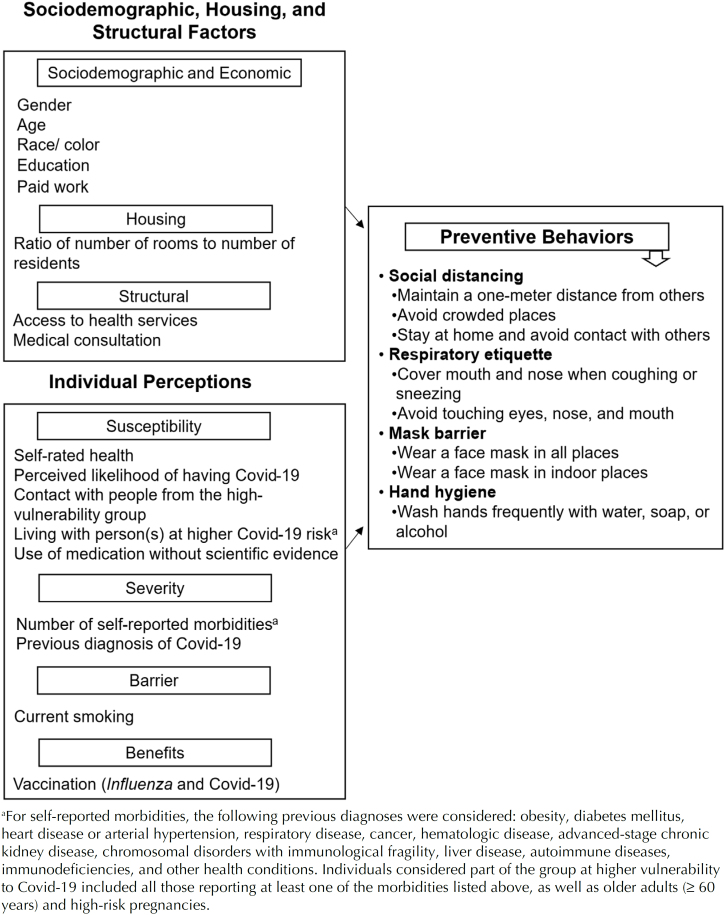
Conceptual model for the assessment of factors associated with Covid-19 preventive behaviors. TQT Covid-19 Study, Brazil, 2023.

The outcome variables were derived from eight Covid-19 preventive behaviors, which were grouped and categorized according to their characteristics: social distancing (maintaining a distance of 1 meter from others, avoiding crowded places, and staying at home); respiratory etiquette (covering the mouth or nose when coughing or sneezing and avoiding touching the eyes and mouth); mask use (wearing a face mask in all settings and a protective mask in enclosed spaces); and hand hygiene (frequent handwashing with soap and water or using alcohol-based sanitizer) (Figure). For each behavior group, the reference category was defined as "no," while the analysis category included participants who answered "yes" to at least one of the questions comprising each outcome.

The sociodemographic, housing, and structural variables included gender (male, female), age (18–39, 40–59, ≥ 60 years), race/color (non-Black [white, yellow, and indigenous], Black [black and brown]), education (elementary school, incomplete or complete high school, higher education or above); paid work in the previous 12 months (no, yes), ratio of household rooms to residents (< 2, ≥ 2); access to health services (exclusively through SUS; or via private services, health plans, or a combination of services), and medical consultation in the previous 12 months (no, yes).

The block of individual perception variables was developed based on the health belief model^5^, focusing on variables indicative of perceived susceptibility and severity, as well as barriers and benefits to adopting preventive behaviors. Perceived susceptibility refers to the belief in one's vulnerability to Covid-19 and included the following variables: self-assessment of health (poor/very poor, fair, very good/good), perceived likelihood of contracting Covid-19 (none/low, moderate/high), contact with individuals with Covid-19 or flu-like symptoms (no, yes), living with person(s) at higher Covid-19 risk (no, yes), and use of medications without scientific evidence for Covid-19 (hydroxychloroquine, chloroquine, and/or ivermectin) (no, yes). Perceived severity refers to the perceived seriousness of the disease and its potential sequelae and included the number of self-reported morbidities (none, one, two or more) and previous diagnosis of Covid-19 (never, once, multiple times). Perceived barriers, defined as potential negative aspects of a specific health action that may impede adherence, were represented by current smoking status (smoker, non-smoker). Perceived benefits, defined as the effectiveness of recommended actions in reducing the risk or severity of Covid-19, included influenza vaccination (no, yes) and Covid-19 vaccination status (up to 2 doses, 1^st^ booster, 2^nd^ booster).

### Analysis

All analyses were stratified by study site (Salvador and Rio de Janeiro). Descriptive statistics were calculated for all variables using frequency measures. The prevalence of each outcome was estimated according to the independent variables. Associations between the independent variables and outcomes were assessed using Pearson's χ² test, and effect sizes were expressed as prevalence ratios (PR) with corresponding 95% confidence intervals (95%CI), estimated using Poisson regression with robust variance. In the multiple regression analysis, variables with p < 0.20 in the bivariate analysis were included in the initial model, and only those with p < 0.05 were retained in the final model. Model comparison was performed using the Akaike and Bayesian information criteria, and the goodness-of-fit of the final model was evaluated using the χ² test. All analyses were conducted using Stata version 16.1.

## RESULTS

Of the 12,883 participants in the TQT Covid-19 Study intervention, 7,941 (61.6%) completed the socio-behavioral questionnaire. This analysis included valid data from 5,476 participants in Salvador and 1,940 in Rio de Janeiro, all aged 18 years or older. Participants under 18 years were excluded due to potential differences in risk perception and limited autonomy in health-related behavioral decisions.

Participants at both sites were predominantly female, aged 18–39 years, self-identified as Black, had completed high school, and had engaged in paid work in the previous 12 months. More than half reported exclusive use of the SUS (66.4% in Salvador and 76.2% in Rio de Janeiro). Differences between the cities were observed in contact with Covid-19 cases (53.2% in Salvador *versus* 43.9% in Rio de Janeiro), prior diagnosis of Covid-19 (36.5 *vs.* 51.1%), and vaccination coverage: in Salvador, 39.2% had received the first booster and 40.4% the second booster, whereas in Rio de Janeiro the figures were 27.3 and 49%, respectively (Table 1).

**Table 1:** Description of sociodemographic, housing, and structural variables and individual perceptions in Salvador/BA (n = 5,476) and in Rio de Janeiro/RJ (n = 1,940). TQT Covid-19 Study, Brazil, 2023.

Sociodemographic, housing, and structural variables		Salvador (n = 5,476)			Rio de Janeiro (n = 1,940)		
		n	%	95%CI	n	%	95%CI
Gender							
	Male	1,579	28.8	27.6–30.0	638	32.9	30.8–35.0
	Female	3,897	71.2	69.9–72.3	1,302	67.1	65.0–69.2
Age (years)							
	18–39	2,541	46.4	45.1–47.7	901	46.5	44.2–48.7
	40–59	2,136	39.0	37.7–40.3	701	36.1	34.0–38.3
	≥ 60	799	14.6	13.7–15.5	338	17.4	15.8–19.2
Race/color							
	Non-Black	559	10.2	9.4–11.0	626	32.3	30.2–34.4
	Black	4,917	89.8	89.0–90.6	1,314	67.7	65.6–69.8
Education							
	Elementary school	1,178	21.5	20.4–22.6	587	30.3	28.2–32.3
	Incomplete/complete high school	2,757	50.4	49.0–51.7	935	48.2	46.0–50.4
	Higher education or more	1,541	28.1	27.0–29.3	418	21.5	19.8–23.4
Paid work							
	Yes	3,552	64.9	63.6–66.1	1,413	72.8	70.8–74.8
	No	1,924	35.1	33.9–36.4	527	27.2	25.2–29.2
Ratio of rooms to residents							
	< 2	2,642	48.2	46.9–49.6	1,216	62.7	60.5–64.8
	≥ 2	2,834	51.8	50.4–53.1	724	37.3	35.2–39.5
Access to health services							
	Exclusively through SUS	3,637	66.4	65.1–67.7	1,478	76.2	74.2–78.0
	Private service, health insurance, or all services	1,839	33.6	32.3–34.8	462	23.8	22.0–25.8
Medical consultation							
	No	1,155	21.1	20.0–22.2	436	22.5	20.7–24.4
	Yes	4,321	78.9	77.8–80.0	1,504	77.5	75.6–79.3
Individual perceptions							
Self-rated health							
	Poor/very poor	763	13.9	13.0–14.9	394	20.3	18.6–22.2
	Fair	2,962	54.1	52.8–55.4	898	46.3	44.1–48.5
	Good/very good	1,748	31.9	30.7–33.2	648	33.4	31.3–35.5
Perception of likelihood of having Covid-19								
	None/low	1,304	23.8	22.7–25.0	812	41.9	40.0–44.1
	Moderate/high	4,167	76.2	75.0–77.3	1,128	58.1	55.9–60.3
Contact with person(s) with Covid-19 or flu symptoms							
	No	2,564	46.8	45.5–48.1	1,088	56.1	53.9–58.3
	Yes	2,911	53.2	51.8–54.5	852	43.9	41.7–46.1
Living with person(s) at higher Covid-19 risk							
	No	3,362	61.4	60.1–62.7	1,272	65.6	63.4–67.7
	Yes	2,114	38.6	37.3–39.9	668	34.4	32.3–36.6
Use of medication without scientific evidence							
	No	4,801	87.7	86.8–88.5	1,742	89.8	88.3–91.1
	Yes	675	12.3	11.5–13.2	198	10.2	8.9–11.6
Number of self-reported morbidities							
	None	3,733	68.2	66.9–69.4	1,256	64.7	62.6–66.8
	One	1,245	22.7	21.6–23.9	516	26.6	24.7–28.6
	Two or more	498	9.1	8.4–9.9	168	8.7	7.5–10.0
Previous Covid-19 diagnosis							
	Never	3,476	63.5	62.2–64.7	949	48.9	46.7–51.1
	One or more times	2,000	36.5	35.2–37.8	991	51.1	48.9–53.3
Current smoking							
	Smoker	339	6.2	5.6–6.9	316	16.3	14.7–18.0
	Non-smoker	5,136	93.8	93.1–94.4	1,624	83.7	82.0–85.3
Influenza vaccine							
	No	1,757	32.1	30.9–33.3	623	32.1	30.1–34.2
	Yes	3,718	67.9	66.7–69.1	1,317	67.9	65.7–70.0
Covid-19 vaccine							
	Up to 2 doses	1,116	20.4	19.3–21.5	460	23.7	21.9–25.7
	1st booster	2144	39.2	37.9–40.5	529	27.3	25.3–29.3
	2nd booster	2,215	40.4	39.2–41.8	951	49.0	46.8–51.2

The most prevalent preventive behaviors for Salvador and Rio de Janeiro were, respectively, hand hygiene (84.9 and 79.1%) and respiratory etiquette (82.7 and 84.3%). Furthermore, a lower prevalence of social distancing (74.9%) was observed in Salvador and of mask use (39.1%) in Rio de Janeiro (Table 2).

**Table 2:** Prevalence of Covid-19 preventive behaviors in Salvador/BA (n = 5,476) and in Rio de Janeiro/RJ (n = 1,940), according to selected variables. TQT Covid-19 Study, Brazil, 2023.

Characteristics		Social distancing		Respiratory etiquette		Mask barrier		Hand hygiene	
		Salvador	Rio de Janeiro	Salvador	Rio de Janeiro	Salvador	Rio de Janeiro	Salvador	Rio de Janeiro
		P (%)	P (%)	P (%)	P (%)	P (%)	P (%)	P (%)	P (%)
Total sample		74.9	58.1	82.7	84.3	79.2	39.1	84.9	79.1
Gender									
	Male	73.0	55.2	79.6	79.9	73.2	32.4	81.1	74.1
	Female	75.7	59.5	84.0	86.4	81.6	42.3	86.4	81.6
Age (years)									
	18–39	69.5	51.4	81.3	84.3	71.8	30.2	82.0	74.8
	40–59	77.7	61.8	84.5	85.2	84.6	41.9	87.5	82.3
	≥ 60	84.7	68.3	82.2	82.2	88.4	56.8	86.9	84.0
Race/color									
	Non-Black	77.8	61.3	84.4	89.3	79.8	39.3	86.8	82.1
	Black	74.6	56.5	82.5	81.9	79.1	39.0	84.6	77.7
Education									
	Elementary school	76.1	61.8	74.3	80.7	79.0	40.5	79.3	75.5
	Incomplete/Complete high school	75.1	57.4	84.1	84.9	80.0	37.7	84.9	80.3
	Higher education or more	73.7	54.3	86.8	87.8	77.9	39.9	89.0	81.6
Paid work									
	Yes	71.8	54.6	83.4	85.0	77.9	38.4	85.8	79.8
	No	80.6	67.5	81.5	82.3	81.6	40.8	83.1	77.2
Ratio of rooms to residents									
	< 2	74.6	57.8	80.5	83.6	78.4	36.8	82.6	77.9
	≥ 2	75.2	58.6	84.8	85.4	80.0	42.8	86.9	81.2
Access to health services									
	Exclusively through SUS	74.2	59.0	81.1	84.5	78.8	38.6	83.0	78.9
	Private service, health insurance, or all services	76.4	55.2	86.0	83.5	80.0	40.5	88.5	79.9
Medical consultation									
	No	73.8	58.0	81.5	81.4	74.3	36.0	83.4	78.9
	Yes	75.2	58.1	83.1	85.1	80.5	40.0	85.3	79.2
Self-rated health									
	Poor/very poor	72.9	52.8	80.7	82.2	73.8	36.3	81.9	80.5
	Fair	75.2	60.5	82.7	84.4	80.0	39.2	84.6	80.7
	Good/very good	75.2	58.0	83.6	85.3	80.1	40.6	86.5	76.1
Perception of likelihood of having Covid-19									
	None/low	80.1	60.7	80.0	82.9	82.3	36.4	84.6	77.6
	Moderate/high	73.3	56.2	83.6	85.3	78.2	41.0	84.9	80.2
Contact with person(s) with Covid-19 or flu symptoms									
	No	76.5	60.8	82.6	82.9	78.0	37.6	84.0	78.0
	Yes	73.6	54.6	82.9	86.0	80.3	41.0	85.7	80.5
Living with person(s) at higher Covid-19 risk									
	No	73.0	56.8	82.1	84.4	77.3	36.6	84.1	78.3
	Yes	78.0	60.5	83.7	84.0	82.2	43.9	86.1	80.7
Use of medication without scientific evidence									
	No	74.6	57.7	83.2	84.1	79.5	38.4	84.9	79.1
	Yes	76.9	61.6	79.6	85.9	77.0	44.9	84.9	79.3
Number of self-reported morbidities									
	None	72.7	55.2	82.5	84.2	76.6	35.0	84.2	78.2
	One	79.0	61.0	83.1	84.1	83.0	45.5	85.9	79.5
	Two or more	81.1	70.2	83.1	85.7	89.0	49.4	87.1	85.1
Previous Covid-19 diagnosis									
	Never	75.5	61.2	81.6	83.3	79.8	37.5	84.0	78.2
	One or more times	74.0	55.1	84.6	85.2	78.2	40.6	86.3	80.0
Current smoking									
	Smoker	69.0	54.1	77.3	77.2	74.0	31.6	78.5	70.9
	Non-smoker	75.3	58.9	83.1	85.6	79.5	40.5	85.3	80.7
Influenza vaccine									
	No	71.4	53.1	80.8	81.5	73.1	27.9	80.8	76.4
	Yes	76.6	60.4	83.6	85.6	82.1	44.3	86.8	80.4
Covid-19 vaccine									
	Up to 2 doses	71.1	49.8	77.1	77.0	72.1	30.2	78.8	70.9
	1st booster	72.9	55.8	83.0	87.5	76.4	31.4	84.5	79.8
	2nd booster	78.7	63.4	85.3	86.0	85.5	47.6	88.3	82.7

P (%): prevalence in percentage; SUS: Unified Health System.

In Salvador, social distancing was more frequent among participants aged 40–59 years (77.7%) and ≥ 60 years (84.7%), those without paid work (80.6%), individuals living with people vulnerable to Covid-19 (78%), and those vaccinated against influenza (76.6%). A moderate to high perceived risk of Covid-19 infection was associated with lower adherence to social distancing (73.3%). Respiratory etiquette was more prevalent among women (84%), participants aged 40–59 years (84.5%), those with secondary (84.1%) or higher education (86.8%), individuals living in higher-density households (84.8%), participants with high perceived risk (83.6%), and those who received Covid-19 booster vaccinations (83% for the first booster and 85.3% for the second booster). Lower adherence was observed among users of medications without scientific evidence (79.6%) (Table 2).

In the same place, mask use was more frequent among women (81.6%), participants aged 40–59 years (84.6%) and ≥60 years (88.4%), those with self-rated health as fair (80%) or good/very good (80.1%), participants with low perceived risk (82.3%), those who had contact with Covid-19 cases (80.3%), lived with vulnerable individuals (82.2%), reported comorbidities (83.1%), received the influenza vaccine (82.1%), and those who had received a Covid-19 booster (76.4% for the first booster and 85.5% for the second booster). Hand hygiene was more common among women (86.4%), participants aged 40–59 years (87.5%) and ≥ 60 years (86.9%), those with secondary (84.9%) or higher education (89%), employed individuals (85.8%), participants living in higher-density households (86.9%), those with better self-rated health (86.5%), those living with vulnerable individuals (86.1%), and participants vaccinated against influenza (86.8%) and Covid-19 (84.5% for the first booster and 88.3% for the second booster) (Table 2).

In Rio de Janeiro, the prevalence of social distancing was higher among participants aged 40–59 years (61.8%) and ≥ 60 years (68.3%), those not working (67.5%), individuals with fair self-rated health (60.5%), participants without contact with Covid-19 cases (60.8%), those without a previous Covid-19 diagnosis (61.2%), and those who had received a Covid-19 booster (55.8% for the first booster and 63.4% for the second booster). Respiratory etiquette was more common among women (86.4%), non-Black participants (89.3%), those with secondary (84.9%) or higher education (87.8%), non-smokers (85.6%), and participants who had received a Covid-19 booster (87.5% for the first booster and 86.0% for the second booster). Mask use was more prevalent among women (42.3%), older participants (40–59 years = 41.9%; ≥ 60 years = 56.8%), those with moderate/high perceived risk of Covid-19 infection (41%), participants vaccinated against influenza (44.3%), and those who had received a Covid-19 booster (31.4% for the first booster and 47.6% for the second booster). Hand hygiene was more frequent among women (81.6%), participants aged 40–59 years (82.3%) and ≥ 60 years (84.0%), those with secondary (80.3%) or higher education (81.6%), and those who had received a Covid-19 booster (79.8% for the first booster and 82.7% for the second booster) (Table 2).

After multiple analysis, in the municipality of Salvador, female gender, older age and education level, perception of the chance of having moderate/high Covid-19 infection, and Covid-19 vaccination booster remained significantly associated with the adoption of most of the behaviors evaluated (Table 3). In Rio de Janeiro, on the other hand, female gender, older age, and Covid-19 vaccination booster were associated with a higher prevalence of adopting most of the preventive behaviors (Table 4).

**Table 3:** Bivariate and multivariate analysis of explanatory variables and Covid-19 preventive behaviors in Salvador/BA (n = 5,476). TQT Covid-19 Study, Brazil, 2023.

Characteristics		Salvador (BA)							
		Social distancing		Respiratory etiquette		Mask barrier		Hand hygiene	
		PR (95%CI)	PR_a_ (95%CI)	PR (95%CI)	PR_a_ (95%CI)	PR (95%CI)	PR_a_ (95%CI)	PR (95%CI)	PR_a_ (95%CI)
Gender									
	Male	1		1	1	1	1	1	1
	Female	**1.04** (1.01–1.07)		**1.05** (1.02–1.08)	**1.05** (1.02–1.08)	**1.11** (1.08–1.15)	**1.09** (1.06–1.14)	**1.06** (1.04–1.09)	**1.06** (1.03–1.09)
Age (years)									
	18–39	1	1	1	1	1	1	1	1
	40–59	**1.12** (1.08–1.16)	**1.12** (1.08–1.16)	**1.04** (1.01–1.07)	**1.04** (1.01–1.07)	**1.18** (1.14–1.21)	**1.14** (1.11–1.18)	**1.07** (1.04–1.09)	**1.06** (1.03–1.09)
	≥ 60	**1.22** (1.17–1.27)	**1.16** (1.11–1.21)	**1.01** (0.97–1.05)	**1.03** (0.99–1.07)	**1.23** (1.19–1.27)	**1.15** (1.10–1.20)	**1.06** (1.02–1.09)	**1.06** (102–1.11)
Race/color									
	Non-Black	1		1		1		1	
	Black	0.96 (0.91–1.01)		0.98 (0.94–1.01)		0.99 (0.95–1.04)		0.98 (0.94–1.01)	
Education									
	Elementary school	1	1	1	1	1		1	1
	Incomplete/Complete high school	0.99 (0.95–1.02)	**1.04** (1.01–1.08)	**1.13** (1.09–1.17)	**1.14** (1.09–1.18)	1.01 (0.98–1.05)		**1.07** (1.04–1.11)	**1.08** (1.04–1.11)
	Higher education or more	0.97 (0.93–1.01)	1.04 (0.99–1.09)	**1.17** (1.12–1.21)	**1.16** (1.11–1.21)	0.99 (0.95–1.03)		**1.12** (1.08–1.16)	**1.11** (1.07–1.15)
Paid work									
	Yes	1	1	1		1		1	1
	No	**1.12** (1.09–1.16)	**1.09** (1.05–1.12)	0.98 (0.95–1.01)		**1.05** (1.02–1.08)		**0.98** (0.94–0.99)	**0.96** (0.94–0.99)
Ratio of rooms to residents									
	< 2	1		1	1	1		1	1
	≥ 2	1.01 (0.98–1.04)		**1.05** (1.03–1.08)	**1.03** (1.01–1.06)	1.02 (0.99–1.05)		**1.05** (1.03–1.08)	**1.04** (1.01–1.06)
Access to health services									
	Exclusively through SUS	1		1		1		1	
	Private service, health insurance, or all services	1.03 (0.99–1.06)		**1.06** (1.03–1.09)		1.01 (0.99–1.04)		**1.06** (1.04–1.09)	
Medical consultation									
	No	1		1		1		1	
	Yes	1.02 (0.98–1.06)		1.02 (0.99–1.05)		**1.08** (1.04–1.12)		1.02 (0.99–1.05)	
Self-rated health									
	Poor/very poor	1		1		1	1	1	1
	Fair	1.03 (0.98–1.08)		1.02 (0.98–1.06)		**1.08** (1.04–1.13)	**1.07** (1.02–1.12)	1.03 (0.99–1.07)	1.03 (0.99–1.07)
	Good/very good	1.03 (0.98–1.09)		1.04 (0.99–1.09)		**1.09** (1.03–1.14)	**1.08** (1.03–1.13)	**1.06** (1.01–1.10)	**1.04** (1.01–1.08)
Perception of likelihood of having Covid-19									
	None/low	1	1	1	1	1	1	1	
	Moderate/high	**0.91** (0.88–0.94)	**0.93** (0.90–0.96)	**1.04** (1.01–1.08)	**1.03** (1.01–1.06)	**0.95** (0.92–0.98)	**0.96** (0.93–0.99)	1.01 (0.98–1.03)	
Contact with person(s) with Covid-19 or flu symptoms									
	No	1		1		1	1	1	
	Yes	**0.96** (0.93–0.99)		1.01 (0.98–1.03)		**1.03** (1.01–1.06)	**1.03** (1.01–1.06)	1.02 (0.99–1.04)	
Living with person(s) at higher Covid-19 risk									
	No	1	1	1		1	1	1	1
	Yes	**1.07** (1.03–1.10)	**1.06** (1.03–1.10)	1.02 (0.99–1.04)		**1.06** (1.03–1.09)	**1.06** (1.03–1.10)	**1.02** (1.01–1.05)	**1.03** (1.01–1.05)
Use of medication without scientific evidence									
	No	1		1	1	1		1	
	Yes	1.03 (0.98–1.08)		**0.96** (0.92–0.99)	**0.95** (0.92–0.99)	0.97 (0.93–1.01)		1.01 (0.97–1.03)	
Number of self-reported morbidities									
	None	1		1		1	1	1	
	One	**1.09** (1.05–1.12)		1.01 (0.99–1.04)		**1.08** (1.05–1.12)	1.01 (0.98–1.04)	1.02 (0.99–1.05)	
	Two or more	**1.11** (1.06–1.17)		1.01 (0.97–1.05)		**1.16** (1.12–1.20)	**1.05** (1.01–1.10)	1.03 (0.99–1.07)	
Previous Covid-19 diagnosis									
	Never	1		1		1		1	
	One or more times	0.98 (0.95–1.01)		**1.04** (1.01–1.06)		0.98 (0.95–1.01)		**1.03** (1.01–1.05)	
Current smoking									
	Smoker	1		1		1		1	
	Non-smoker	**1.09** (1.01–1.17)		**1.07** (1.01–1.14)		**1.07** (1.01–1.15)		**1.09** (1.03–1.15)	
Influenza vaccine									
	No	1	1	1		1	1	1	1
	Yes	**1.07** (1.04–1.11)	**1.04** (1.01–1.08)	**1.04** (1.01–1.06)		**1.12** (1.09–1.16)	**1.06** (1.03–1.10)	**1.07** (1.05–1.10)	**1.05** (1.02–1.07)
Covid-19 vaccine									
	Up to 2 doses	1		1	1	1	1	1	1
	1st booster	1.02 (0.98–1.07)		**1.07** (1.04–1.12)	**1.05** (1.01–1.09)	**1.06** (1.01–1.11)	1.03 (0.98–1.07)	**1.07** (1.03–1.11)	**1.04** (1.01–1.08)
	2nd booster	**1.11** (1.06–1.15)		**1.11** (1.07–1.15)	**1.07** (1.04–1.12)	**1.18** (1.14–1.23)	**1.08** (1.03–1.13)	**1.12** (1.08–1.16)	**1.06** (1.02–1.10)

PR: prevalence ratio; PR_a_: adjusted prevalence ratio; 95%CI: 95% confidence interval.

Note: significant results (p < 0.05) are shown in bold.

**Table 4:** Bivariate and multivariate analysis of explanatory variables and Covid-19 preventive behaviors in Rio de Janeiro (RJ) (n = 1,940). TQT Covid-19 Study, Brazil, 2023.

Characteristics		Rio de Janeiro (RJ)							
		Social distancing		Respiratory etiquette		Respiratory etiquette		Hand hygiene	
		PR (95%CI)	PR_a_ (95%CI)	PR (95%CI)	PR_a_ (95%CI)	PR (95%CI)	PR_a_ (95%CI)	PR (95%CI)	PR_a_ (95%CI)
Gender									
	Male	1		1	1	1	1	1	1
	Female	1.08 (0.99–1.17)		**1.08** (1.03–1.13)	**1.07** (1.02–1.12)	**1.30** (1.15–1.48)	**1.23** (1.08–1.40)	**1.10** (1.04–1.16)	**1.09** (1.03–1.15)
Age (years)									
	18–39	1	1	1		1	1	1	1
	40–59	**1.20** (1.10–1.31)	**1.19** (1.09–1.30)	1.01 (0.97–1.05)		**1.39** (1.22–1.58)	**1.29** (1.12–1.47)	**1.10** (1.04–1.16)	**1.10** (1.04–1.16)
	≥ 60	**1.33** (1.21–1.46)	**1.18** (1.06–1.31)	0.97 (0.92–1.03)		**1.88** (1.64–2.16)	**1.64** (1.42–1.89)	**1.12** (1.06–1.19)	**1.13** (1.06–1.21)
Race/color									
	Non-Black	1		1	1	1		1	
	Black	**0.92** (0.85–0.99)		**0.92** (0.88–0.95)	**0.93** (0.89–0.96)	0.99 (0.88–1.12)		**0.95** (0.90–0.99)	
Education									
	Elementary school	1		1	1	1		1	1
	Incomplete/Complete high school	0.93 (0.85–1.01)		**1.05** (1.01–1.10)	**1.05** (1.01–1.10)	0.93 (0.82–1.06)		**1.06** (1.01–1.13)	**1.10** (1.04–1.17)
	Higher education or more	**0.88** (0.79–0.98)		**1.09** (1.03–1.15)	1.05 (0.99–1.11)	0.98 (0.84–1.15)		**1.08** (1.01–1.15)	**1.10** (1.04–1.19)
Paid work									
	Yes	1	1	1		1		1	
	No	**1.24** (1.15–1.34)	**1.17** (1.08–1.27)	0.97 (0.93–1.01)		1.06 (0.94–1.20)		0.97 (0.92–1.02)	
Ratio of rooms to residents									
	< 2	1		1		1		1	
	≥ 2	1.01 (0.94–1.09)		1.02 (0.98–1.06)		**1.16** (1.04–1.30)		1.04 (0.99–1.09)	
Access to health services									
	Exclusively through SUS	1		1		1		1	
	Private service, health insurance, or all services	0.93 (0.85–1.03)		0.99 (0.94–1.03)		1.05 (0.92–1.20)		1.01 (0.96–1.07)	
Medical consultation									
	No	1		1		1		1	
	Yes	1.01 (0.91–1.10)		1.04 (0.99–1.10)		1.11 (0.96–1.28)		1.01 (0.95–1.06)	
Self-rated health									
	Poor/very poor	1	1	1		1		1	
	Fair	**1.14** (1.03–1.27)	**1.14** (1.03–1.27)	1.03 (0.97–1.08)		1.08 (0.93–1.26)		1.01 (0.95–1.06)	
	Good/very good	1.10 (0.98–1.23)	1.10 (0.99–1.23)	1.04 (0.98–1.10)		1.12 (0.95–1.31)		0.95 (0.89–1.01)	
Perception of likelihood of having Covid-19									
	None/low	1		1		1	1	1	
	Moderate/high	**0.93** (0.86–0.99)		1.03 (0.99–1.07)		**1.12** (1.01–1.26)	**1.13** (1.01–1.27)	1.03 (0.99–1.08)	
Contact with person(s) with Covid-19 or flu symptoms									
	No	1	1	1		1		1	
	Yes	**0.90** (0.83–0.97)	**0.92** (0.85–0.99)	**1.04** (1.01–1.08)		1.09 (0.97–1.22)		1.03 (0.99–1.08)	
Living with person(s) at higher Covid-19 risk									
	No	1		1		1		1	
	Yes	1.06 (0.98–1.15)		0.99 (0.95–1.04)		**1.20** (1.07–1.34)		1.03 (0.98–1.08)	
Use of medication without scientific evidence									
	No	1		1		1		1	
	Yes	1.07 (0.95–1.20)		1.02 (0.96–1.08)		1.17 (0.99–1.38)		1.01 (0.93–1.08)	
Number of self-reported morbidities									
	None	1		1		1		1	
	One	**1.10** (1.01–1.20)		0.99 (0.96–1.04)		**1.30** (1.15–1.47)		1.01 (0.96–1.07)	
	Two or more	**1.27** (1.14–1.42)		1.02 (0.95–1.09)		**1.41** (1.19–1.67)		**1.09** (1.01–1.17)	
Previous Covid-19 diagnosis									
	Never	1	1	1		1		1	
	One or more times	**0.90** (0.83–0.97)	**0.90** (0.84–0.98)	1.02 (0.98–1.06)		1.08 (0.97–1.21)		1.02 (0.98–1.07)	
Current smoking									
	Smoker	1		1	1	1		1	
	Non-smoker	1.09 (0.97–1.21)		**1.11** (1.04–1.18)	**1.08** (1.02–1.15)	**1.28** (1.08–1.52)		**1.14** (1.06–1.23)	
Influenza vaccine									
	No	1		1		1	1	1	
	Yes	**1.14** (1.04–1.24)		**1.05** (1.01–1.10)		**1.59** (1.38–1.83)	**1.32** (1.14–1.53)	1.05 (0.99–1.11)	
Covid-19 vaccine									
	Up to 2 doses	1	1	1	1	1	1	1	1
	1st booster	1.12 (0.99–1.26)	**1.13** (1.01–1.27)	**1.14** (1.07–1.21)	**1.12** (1.06–1.19)	1.04 (0.86–1.25)	0.97 (0.81–1.17)	**1.13** (1.05–1.21)	**1.11** (1.04–1.19)
	2nd booster	**1.27** (1.15–1.41)	**1.21** (1.09–1.35)	**1.12** (1.06–1.18)	**1.10** (1.04–1.16)	**1.58** (1.35–1.84)	**1.21** (1.03–1.43)	**1.17** (1.09–1.25)	**1.12** (1.04–1.19)

PR: prevalence ratio; PR_a_: adjusted prevalence ratio; 95%CI: 95% confidence interval.

Note: significant results (p < 0.05) are shown in bold.

## DISCUSSION

This study found that respiratory etiquette and hand hygiene were the most commonly adopted preventive behaviors during the regular period of the pandemic in both Salvador and Rio de Janeiro. Female gender, older age, higher education level, and receipt of recommended vaccines or booster doses were the factors most strongly associated with increased prevalence of the evaluated preventive behaviors, which showed changes after the peak of the pandemic.

The findings indicated that face mask use and physical distancing were less frequently adopted during the regular period of the pandemic, particularly in Rio de Janeiro. A previous study conducted in Manguinhos during the pandemic peak reported low adherence to social distancing and high compliance with mask use^13^. Similarly, the 2020 EpiCovid-19 Brazil study found high prevalences of social distancing and mask use when leaving home, both among individuals with and without chronic non-communicable diseases^9^. It is important to note that data collection occurred at different times in Salvador and Rio de Janeiro; in the latter, it coincided with greater control of Covid-19, widespread vaccination, and continued after the official end of the pandemic. These temporal differences, in addition to methodological variations, may explain the observed differences in behavior prevalence.

Female participants in this study adopted more Covid-19 preventive behaviors, consistent with findings from other regions^14-18^. A meta-analysis indicated that women are approximately 50% more likely than men to implement non-pharmacological preventive measures^14^. During the Covid-19 pandemic, care behaviors differed by gender, with women more likely to perceive the pandemic as a serious health threat and to comply with restrictive measures^15^. In Chile, women reported higher levels of concern and fear regarding the risk of infection and virus transmission, leading to greater adoption of preventive behaviors^16^. Data from 175 countries further demonstrated that women exhibited higher adherence to health protection behaviors against Covid-19^17^. These findings underscore the importance of considering gender in the design of risk communication campaigns.

Older age was associated with higher adoption of most evaluated preventive behaviors, except for respiratory etiquette in Rio de Janeiro. Covid-19 infection is known to have greater severity and higher mortality among older adults^19^, which may contribute to an increased perceived threat in this population. This finding aligns with other studies reporting that older individuals have a heightened perception of Covid-19 risk^20^ and are more likely to adopt protective behaviors^21^.

Higher education levels were associated with greater adoption of respiratory etiquette and hand hygiene, consistent with findings from other studies^20-23^. In South Africa, individuals with 8 or more years of schooling and those with higher incomes demonstrated higher perceived risk of Covid-19^20^. Ozdemir et al.^21^ reported that higher education increased the frequency of adopting various preventive behaviors. Fang et al.^22^ found that education positively influenced the adoption of preventive behaviors at the pandemic peak; however, unlike our results, they did not observe this effect during periods of regular epidemic activity.

Not having engaged in paid work in the previous 12 months was associated with a higher prevalence of social distancing in both sites. Szwarcwald et al.^7^ reported that employed individuals face greater difficulty maintaining social distancing, which may reduce adherence to certain preventive behaviors. Similarly, an analysis of sexual and gender minorities in Brazil during the early months of the pandemic found that increased vulnerability was linked to difficulty maintaining social distancing, likely because these individuals needed to leave home for work, as remote work options were limited for these groups^23^.

Among the variables related to susceptibility to Covid-19, a moderate to high perceived risk of infection influenced the adoption of certain preventive behaviors, either increasing or decreasing adherence. This variable may exhibit a bidirectional relationship with behavior, as individuals unable to comply with specific measures may perceive themselves at greater risk of infection. This could explain the lower prevalence of social distancing and mask use observed in Salvador. It is also important to note that, during the regular period of the pandemic, some preventive measures were relaxed due to the benefits associated with increased vaccination coverage^24^. Additionally, data collection in Rio de Janeiro occurred within a more consolidated and organized public health system.

Despite the relaxation of preventive measures and the reduced incidence of Covid-19, the disease continues to pose a health threat^24^. Persistent fear of virus exposure may have increased adherence to social distancing, mask use, and hand hygiene in Salvador among participants living with individuals vulnerable to Covid-19. Similarly, Silva et al.^25^, in the state of Ceará, reported higher adherence to social distancing among participants concerned about exposing family members to the risk of infection.

The use of medications without scientific evidence for the treatment or prevention of Covid-19 was infrequent in this study; however, it was negatively associated with adherence to respiratory etiquette in Salvador. It is important to note that the study was conducted during a period of political transition in Brazil and during the regular phase of the pandemic, characterized by lower mortality and reduced incidence of new infections. Under the previous federal administration, the Ministry of Health faced severe financial constraints, and the public health system was disorganized, under-resourced, and undergoing a process of dismantling^26^. During this period, preventive measures were discouraged, while unproven interventions, such as the use of medications for Covid-19, were promoted.

In the first year of the current administration (2023), health strategies aimed at restructuring the Ministry of Health focused on strengthening the technical and scientific management of SUS, restoring the National Immunization Program, and intensifying the Covid-19 response^27^. One such strategy was ensuring continuity of population immunization against Covid-19 and influenza, which was reflected in high adherence to the Covid-19 booster dose among participants at both study sites. Receipt of these vaccines was associated with greater adoption of the evaluated preventive behaviors in Salvador and Rio de Janeiro.

This study has some limitations. Data were self-reported and therefore subject to information, recall, and social desirability biases, which may have led to overestimation of the adoption of certain health behaviors. Comparison of the study samples with the population from the most recent demographic census revealed differences in gender, age, and race, with a higher proportion of women, Black individuals, and a median age exceeding that of the general population^28^. Given that a convenience sample was used, with voluntary participation based on health unit attendance, these differences were expected. Consequently, the sampling approach may have resulted in a sample that is not fully representative of the evaluated regions, as it included only individuals with effective access to health services. Nevertheless, the diverse strategies employed to engage the target population likely increased the inclusion of participants from multiple social strata.

A key strength of this study is that it was conducted during the regular period of the pandemic and focused on socially vulnerable populations, in contrast to most research on Covid-19 preventive behaviors. This approach allowed the identification of behaviors that were maintained after the pandemic peak.

These findings make a significant contribution to public health by identifying barriers and facilitators to adherence to Covid-19 preventive behaviors, thereby supporting efforts to reduce inequalities in access to healthcare. Moreover, they provide valuable insights to promote societal awareness of epidemic prevention and to strengthen response capacity among vulnerable populations. Such efforts should also engage healthcare networks in ongoing community education, with the aim of empowering the population, combating misinformation, and fostering a culture of prevention and public health care.

In conclusion, this study identified high adherence to preventive behaviors during the regular period of the pandemic. Maintenance of these behaviors exceeded expectations, particularly for respiratory etiquette and hand hygiene, whereas mask use and social distancing declined, most notably in Rio de Janeiro. Female gender, older age, higher education levels, and vaccination status were associated with increased adoption of Covid-19 preventive behaviors.

## Data Availability

The datasets generated and/or analyzed in this study are not publicly available, as they are part of a larger project with ongoing analyses. However, they can be made available by the corresponding author upon reasonable request.
