# Supplementary material for: Adoption of Covid-19 preventive behaviors in the community: Salvador and Rio de Janeiro
Source: Rev Saude Publica. 2026 May 1;60(Suppl 1):e3s. doi: 10.11606/s1518-8787.2026060006406 (PMC13143268; doi:10.11606/s1518-8787.2026060006406)
Supplement: Translation

# Translation: Adoção de comportamentos preventivos à Covid-19 na comunidade: Salvador e Rio de Janeiro

**Authors:** Gabriele Amorim, Danielle Souto de Medeiros, Fabiane Soares, Laio Magno, Thaís Regis Aranha Rossi, Thiago Silva Torres, Valdiléa Gonçalves Veloso, Débora Castanheira, Inês Dourado

**Affiliations:** I Universidade Federal da Bahia. Instituto Multidisciplinar de Saúde. Programa de Pós-Graduação em Saúde Coletiva. Vitória da Conquista, BA, Brasil; II Universidade Federal da Bahia. Instituto de Saúde Coletiva. Salvador, BA, Brasil; III Universidade do Estado da Bahia. Departamento de Ciências da Vida. Salvador, BA, Brasil; IV Fundação Oswaldo Cruz. Instituto Gonçalo Moniz. Salvador, BA, Brasil; V Fundação Oswaldo Cruz. Instituto Nacional de Infectologia Evandro Chagas. Rio de Janeiro, RJ, Brasil

**Keywords:** COVID-19, Comportamento, Prevenção de Doenças, Pandemias

## Abstract

**OBJETIVO:**

Descrever a prevalência da adoção de comportamentos preventivos à Covid-19 e avaliar os fatores associados entre usuários de unidades de saúde em Salvador e no Rio de Janeiro.

**MÉTODOS:**

Estudo transversal, realizado entre julho de 2022 e julho de 2023. A adoção de comportamentos preventivos foi avaliada com base em oito comportamentos, agrupados nos desfechos: distanciamento social; etiqueta respiratória; barreira por máscara; higienização das mãos; características sociodemográficas, habitacionais, estruturais e percepções individuais. Todas as análises foram estratificadas pelo sítio do estudo. Análises bivariadas e múltiplas foram feitas por regressão de Poisson com variância robusta.

**RESULTADOS:**

Foram avaliados 5.476 participantes de Salvador e 1.940 do Rio de Janeiro. Os comportamentos preventivos mais prevalentes foram a etiqueta respiratória (82,7 e 84,3%) e a higienização das mãos (84,9 e 79,1%), respectivamente. Em Salvador, a idade permaneceu associada a todos os comportamentos avaliados. No Rio de Janeiro, a adoção dos comportamentos preventivos aumentou entre os que utilizaram o reforço da vacina de Covid-19. Em ambas as cidades, indivíduos com idades entre 40 e 59, com 60 anos ou mais e aqueles que não trabalhavam apresentaram maior adesão ao distanciamento social. A prática da etiqueta respiratória foi mais prevalente entre mulheres, pessoas com ensino médio e que receberam reforço vacinal para Covid-19. O uso de máscara e a higienização das mãos estiveram associados ao sexo feminino, à maior idade e ao reforço vacinal nas duas localidades. O uso de máscara também foi mais frequente entre os que se vacinaram contra influenz*a*, enquanto a higienização das mãos mostrou associação com maior escolaridade.

**CONCLUSÕES:**

Esses achados reforçam a importância de políticas públicas que influenciem na manutenção dos comportamentos preventivos e na sensibilização da prevenção de novas epidemias, especialmente entre homens, pessoas mais jovens, com menor escolaridade e que não utilizam as vacinas oferecidas ou as suas doses de reforço.

## INTRODUÇÃO

A Covid-19 tornou-se um problema de saúde pública intrincado, com repercussões no comportamento populacional. Autoridades mundiais estabeleceram medidas de saúde pública para minimizar o colapso dos sistemas de saúde, nos moldes de combate a epidemias infecciosas, com ações preventivas e curativas^1^. O distanciamento físico, uso de máscara, adoção de medidas de etiqueta respiratória e higiene, redução da circulação de pessoas e quarentena (caracterizada por limitação de movimentação ou separação de indivíduos por um período determinado), constituíram as principais medidas recomendadas para minimizar a propagação comunitária da Covid-19, associadas a políticas de proteção social^1,2^.

No Brasil, a instabilidade política inviabilizou uma gestão unificada de enfrentamento à epidemia de Covid-19 entre as diferentes esferas de poder^3^. O presidente, à época da pandemia, minimizou a importância da adoção de comportamentos preventivos, influenciando negativamente a adesão populacional. O "efeito Bolsonaro" afetou negativamente o comportamento da população brasileira, repercutindo em maiores taxas de mortalidade e menor adesão ao distanciamento físico, à vacinação contra a Covid-19 e às demais vacinas preconizadas pelo Programa Nacional de Imunização^4^.

As medidas preventivas de enfrentamento à Covid-19 demandaram a incorporação e/ou adequação de comportamentos em saúde, cuja aceitabilidade ou rejeição sofrem influências a nível individual e das inter-relações interpessoais e coletiva. A suscetibilidade e gravidade percebidas, os benefícios e as barreiras são construtos que embasam a tomada de decisão. Esses, influenciados por características sociodemográficas, habitacionais e estruturais, podem ser determinantes para a aceitação ou recusa da adoção de comportamentos em saúde^5^.

Grande parte das pesquisas anteriores sobre as mudanças comportamentais ocasionadas pela Covid-19 concentrou-se no pico pandêmico, entre 2020 e 2021, levando a diferentes comportamentos adaptativos e desadaptativos^6-9^. Entretanto, pouco se sabe sobre a manutenção desses comportamentos após esse período, sobretudo em populações de áreas socioeconomicamente vulneráveis.

Este estudo teve como objetivo descrever a prevalência da adoção de comportamentos preventivos à Covid-19 e avaliar os fatores associados entre usuários de unidades de saúde em Salvador/BA e no Rio de Janeiro/RJ.

## MÉTODOS

Este estudo teve delineamento transversal, realizado no período de julho de 2022 a julho de 2023, com usuários de unidades de saúde situadas em regiões de maior vulnerabilidade socioeconômica em Salvador, no distrito sanitário Cabula-Beiru, e no Rio de Janeiro, em Manguinhos. Essas regiões são marcadas pelo enfrentamento de desafios sociais, com problemas relacionados à infraestrutura, à segurança e ao acesso a serviços públicos essenciais^10,11^. Trata-se de um recorte da pesquisa "Ampliação das estratégias de testagem, quarentena, saúde digital e telemonitoramento para o enfrentamento da pandemia de Covid-19 no Brasil", também denominado Estudo TQT Covid-19^12^.

O Estudo TQT Covid-19 foi conduzido de acordo com as diretrizes da resolução da Comissão de Ética em Pesquisa (CEP) do Brasil. O protocolo foi aprovado pelos CEP da Organização Mundial da Saúde (CERC.0128A e CERC.0128B) e Institucionais locais (Instituto de Saúde Coletiva / Universidade Federal da Bahia – ISC/UFBA/Salvador: n.º 53844121.4.1001.5030; e no Rio de Janeiro – Instituto Nacional de Infectologia/Fundação Oswaldo Cruz – INI/Fiocruz: n.º 53844121.4.3001.5240, Escola Nacional de Saúde Pública Sérgio Arouca / Fundação Oswaldo – ENSP/Fiocruz: n.º 53844121.4.3001.5240, e Secretaria Municipal de Saúde/Rio de Janeiro – SMS/RJ: n.º 53844121.4.3002.5279). Todos os participantes concordaram em participar da pesquisa e assinaram o Termo de Consentimento Livre e Esclarecido.

A amostra utilizada foi caracterizada como não probabilística, por conveniência, compreendendo usuários do Sistema Único de Saúde (SUS) residentes nas regiões estudadas. Para a construção deste artigo foram avaliados os dados válidos de participantes com idade ≥ 18 anos.

Nas unidades de saúde foram ofertados testes rápidos para Covid-19 a todas as pessoas que tivessem indicação, de acordo com critérios epidemiológicos. Os participantes foram convidados a realizar a testagem e a participar da pesquisa por meio de várias estratégias de sensibilização: divulgação na internet e redes sociais, rádios comunitárias e podcasts, recrutamento por agentes de saúde, ações no Programa Saúde na Escola, além de mobilização de instituições religiosas e de organizações da sociedade civil.

Inicialmente eram realizados o teste rápido de Covid-19 e a aplicação de um questionário sociocomportamental no momento do atendimento no serviço de saúde, enquanto o resultado do teste era aguardado. O questionário foi composto por sete blocos: dados sociodemográficos e de habitação; comorbidades; acesso e utilização de serviços de saúde; infecção e testes anteriores para Covid-19; vacinação; comportamentos, atitudes e práticas de prevenção da Covid-19 e automedicação, percepção de gravidade e risco de infecção; e autoteste.

A coleta de dados no município de Salvador aconteceu entre julho de 2022 e abril de 2023; já no Rio de Janeiro, ocorreu entre novembro de 2022 e julho de 2023. A equipe de pesquisa, formada por profissionais de saúde e agentes comunitários, foi previamente treinada.

Para avaliar os fatores associados aos comportamentos de prevenção à Covid-19, foi desenvolvido um modelo conceitual composto por variáveis sociodemográficas, habitacionais e estruturais, e outras de percepções individuais (Figura).

**Figura:** Modelo conceitual para a avaliação de fatores associados aos comportamentos preventivos à Covid-19. Estudo TQT Covid-19, Brasil, 2023.

As variáveis de desfechos foram construídas a partir de oito comportamentos preventivos à Covid-19, agrupados e categorizados segundo as suas características: distanciamento social (manter distância de 1 metro das pessoas, evitar lugares com muitas pessoas e ficar em casa); etiqueta respiratória (cobrir a boca ou nariz ao tossir ou espirrar e evitar tocar os olhos e boca); barreiras por máscara (usar máscara facial em todos os ambientes e máscara de proteção em lugares fechados); higienização das mãos (lavar as mãos frequentemente com água e sabão ou álcool) (Figura). Para a criação de cada grupo de comportamento, a categoria de referência foi "não", enquanto a categoria de análise foi composta pela resposta "sim" a pelo menos uma das questões que compuseram cada desfecho.

As variáveis sociodemográficas, habitacionais e estruturais foram: sexo (masculino e feminino), idade (18–39, 40–59, ≥ 60 anos), raça/cor (não negra [branca, amarela e indígena], negra [preta e parda]), escolaridade (ensino fundamental, ensino médio completo e incompleto, ensino superior e mais); trabalho remunerado nos últimos 12 meses (não, sim), relação número de cômodos no domicílio por número de morador (< 2, ≥ 2); acesso aos serviços de saúde (exclusivamente pelo SUS; e serviço privado, plano de saúde ou todos os serviços), consulta médica nos últimos 12 meses (não, sim).

O bloco de variáveis de percepções individuais foi desenhado com base no modelo de crenças em saúde^5^, fundamentado na identificação de variáveis indicadoras da suscetibilidade e gravidade percebidas, de barreiras e benefícios à adoção do comportamento. A suscetibilidade se refere à crença da vulnerabilidade ao adoecimento pela Covid-19, e foram considerados nesse grupo: autoavaliação da saúde (ruim/muito ruim, regular, muito boa/boa), percepção da chance de ter Covid-19 (nenhuma/baixa, moderada/alta), ser contatante de pessoa(s) com Covid-19 ou sintomas gripais (não, sim), residir com pessoa(s) do grupo em maior vulnerabilidade à Covid-19 (não, sim) e uso de medicamento sem comprovação científica (hidroxicloroquina, cloroquina e/ou ivermectina) para a Covid-19 (não, sim). A gravidade é a percepção de quão sérias são a condição e as suas possíveis sequelas, sendo incluídas as variáveis: número de morbidades autorreferidas (nenhuma, uma, duas e mais) e diagnóstico prévio de Covid-19 (nenhuma vez, uma vez e mais). As barreiras percebidas são potenciais aspectos negativos de uma ação de saúde específica e podem atuar como impedimentos para a realização de comportamentos recomendados. Neste estudo, considerou-se o tabagismo atual (fumante, não fumante). Os benefícios percebidos consistem na eficácia da ação recomendada para reduzir o risco ou a gravidade do impacto da Covid-19, sendo incluídas as vacinações contra influenza (não, sim) e contra a Covid-19 (até 2 doses, 1º reforço, 2º reforço).

### Análises

Todas as análises foram estratificadas segundo o sítio do estudo (Salvador e Rio de Janeiro). Foi realizada análise descritiva para as variáveis estudadas por medidas de frequência. As prevalências de cada desfecho foram estimadas para as variáveis independentes. A associação entre essas variáveis e os desfechos foi avaliada pelo teste χ^2^ de Pearson e a magnitude, estimada pela Razão de Prevalência (RP), com seus respectivos intervalos de confiança de 95% (IC95%), por regressão de Poisson com variância robusta. A análise múltipla incluiu no modelo inicial as variáveis com p < 0,20 na análise bivariada, e apenas aquelas com p < 0,05 permaneceram no modelo final. Os critérios de Akaike e Bayesiano foram utilizados para a comparação entre os modelos e o teste qui-quadrado (bondade de ajuste), para a adequação do modelo final. As análises foram conduzidas no Stata 16.1.

## RESULTADOS

Dos 12.883 participantes da intervenção do Estudo TQT Covid-19, 7.941 (61,6%) responderam ao questionário sociocomportamental. Esta análise considerou dados válidos de 5.476 participantes de Salvador e 1.940 do Rio de Janeiro, com 18 anos ou mais. Indivíduos menores de 18 anos foram excluídos por possíveis diferenças na percepção de risco e por não apresentarem autonomia plena para decisões comportamentais em saúde.

Os participantes de ambos os sítios foram em maioria do sexo feminino, com idade entre 18 e 39 anos, que se autodeclararam de raça negra, cursaram ensino médio e trabalharam nos últimos 12 meses. Mais da metade relatou acesso exclusivo ao SUS (66,4% em Salvador e 76,2% no Rio de Janeiro). Houve diferenças entre as cidades quanto ao contato com casos de Covid-19 (53,2% em Salvador e 43,9% no Rio de Janeiro), diagnóstico prévio (36,5 e 51,1%) e número de doses recebidas: em Salvador, 39,2% haviam tomado o 1º reforço e 40,4%, o 2º; no Rio de Janeiro, 27,3 e 49%, respectivamente (Tabela 1).

**Tabela 1:** Descrição das variáveis sociodemográficas, habitacionais e estruturais e das percepções individuais em Salvador/BA (n = 5.476) e no Rio de Janeiro/RJ (n = 1.940). Estudo TQT Covid-19, Brasil, 2023.

Variáveis sociodemográficas, habitacionais e estruturais		Salvador (n = 5.476)			Rio de Janeiro (n = 1.940)		
		n	%	IC95%	n	%	IC95%
Sexo							
	Masculino	1.579	28,8	27,6–30,0	638	32,9	30,8–35,0
	Feminino	3.897	71,2	69,9–72,3	1.302	67,1	65,0–69,2
Idade (anos)							
	18–39	2.541	46,4	45,1–47,7	901	46,5	44,2–48,7
	40–59	2.136	39,0	37,7–40,3	701	36,1	34,0–38,3
	≥ 60	799	14,6	13,7–15,5	338	17,4	15,8–19,2
Raça/cor							
	Não negra	559	10,2	9,4–11,0	626	32,3	30,2–34,4
	Negra	4.917	89,8	89,0–90,6	1.314	67,7	65,6–69,8
Escolaridade							
	Ensino fundamental	1.178	21,5	20,4–22,6	587	30,3	28,2–32,3
	Ensino médio completo e incompleto	2.757	50,4	49,0–51,7	935	48,2	46,0–50,4
	Ensino superior e mais	1.541	28,1	27,0–29,3	418	21,5	19,8–23,4
Trabalho remunerado							
	Sim	3.552	64,9	63,6–66,1	1.413	72,8	70,8–74,8
	Não	1.924	35,1	33,9–36,4	527	27,2	25,2–29,2
Relação nº de cômodos por nº de moradores							
	< 2	2.642	48,2	46,9–49,6	1.216	62,7	60,5–64,8
	≥ 2	2.834	51,8	50,4–53,1	724	37,3	35,2–39,5
Acesso aos serviços de saúde							
	Exclusivamente pelo SUS	3.637	66,4	65,1–67,7	1.478	76,2	74,2–78,0
	Serviço privado, plano de saúde ou todos os serviços	1.839	33,6	32,3–34,8	462	23,8	22,0–25,8
Consulta médica							
	Não	1.155	21,1	20,0–22,2	436	22,5	20,7–24,4
	Sim	4.321	78,9	77,8–80,0	1.504	77,5	75,6–79,3
Percepções individuais							
Autoavaliação da saúde							
	Ruim/muito ruim	763	13,9	13,0–14,9	394	20,3	18,6–22,2
	Regular	2.962	54,1	52,8–55,4	898	46,3	44,1–48,5
	Muito boa/boa	1.748	31,9	30,7–33,2	648	33,4	31,3–35,5
Percepção da chance de ter Covid-19							
	Nenhuma/baixa	1.304	23,8	22,7–25,0	812	41,9	40,0–44,1
	Moderada/alta	4.167	76,2	75,0–77,3	1.128	58,1	55,9–60,3
Contatante de pessoa(s) com Covid-19 ou sintomas gripais							
	Não	2.564	46,8	45,5–48,1	1.088	56,1	53,9–58,3
	Sim	2.911	53,2	51,8–54,5	852	43,9	41,7–46,1
Residir com pessoa(s) do grupo em maior vulnerabilidade à Covid-19							
	Não	3.362	61,4	60,1–62,7	1.272	65,6	63,4–67,7
	Sim	2.114	38,6	37,3–39,9	668	34,4	32,3–36,6
Uso de medicamento sem comprovação científica							
	Não	4.801	87,7	86,8–88,5	1.742	89,8	88,3–91,1
	Sim	675	12,3	11,5–13,2	198	10,2	8,9–11,6
Número de morbidades autorreferidas							
	Nenhuma	3.733	68,2	66,9–69,4	1.256	64,7	62,6–66,8
	Uma	1.245	22,7	21,6–23,9	516	26,6	24,7–28,6
	Duas ou mais	498	9,1	8,4–9,9	168	8,7	7,5–10,0
Diagnóstico prévio de Covid-19							
	Nenhuma vez	3.476	63,5	62,2–64,7	949	48,9	46,7–51,1
	Uma ou mais vezes	2.000	36,5	35,2–37,8	991	51,1	48,9–53,3
Tabagismo atual							
	Fumante	339	6,2	5,6–6,9	316	16,3	14,7–18,0
	Não fumante	5.136	93,8	93,1–94,4	1.624	83,7	82,0–85,3
Vacina influenza							
	Não	1.757	32,1	30,9–33,3	623	32,1	30,1–34,2
	Sim	3.718	67,9	66,7–69,1	1.317	67,9	65,7–70,0
Vacina Covid-19							
	Até 2 doses	1.116	20,4	19,3–21,5	460	23,7	21,9–25,7
	1º reforço	2144	39,2	37,9–40,5	529	27,3	25,3–29,3
	2º reforço	2.215	40,4	39,2–41,8	951	49,0	46,8–51,2

Os comportamentos preventivos mais prevalentes para Salvador e Rio de Janeiro foram, respectivamente, a higienização das mãos (84,9 e 79,1%) e a etiqueta respiratória (82,7 e 84,3%). Ainda, observou-se menor prevalência do distanciamento social (74,9%) em Salvador e da barreira por máscara (39,1%) no Rio de Janeiro (Tabela 2).

**Tabela 2:** Prevalências dos comportamentos preventivos à Covid-19 em Salvador/BA (n = 5.476) e no Rio de Janeiro/RJ (n = 1.940), segundo variáveis selecionadas. Estudo TQT Covid-19, Brasil, 2023.

Variáveis		Distanciamento social		Etiqueta respiratória		Barreira por máscara		Higienização das mãos	
		Salvador	Rio de Janeiro	Salvador	Rio de Janeiro	Salvador	Rio de Janeiro	Salvador	Rio de Janeiro
		P (%)	P (%)	P (%)	P (%)	P (%)	P (%)	P (%)	P (%)
Amostra total		74,9	58,1	82,7	84,3	79,2	39,1	84,9	79,1
Sexo									
	Masculino	73,0	55,2	79,6	79,9	73,2	32,4	81,1	74,1
	Feminino	75,7	59,5	84,0	86,4	81,6	42,3	86,4	81,6
Idade (anos)									
	18–39	69,5	51,4	81,3	84,3	71,8	30,2	82,0	74,8
	40–59	77,7	61,8	84,5	85,2	84,6	41,9	87,5	82,3
	≥ 60	84,7	68,3	82,2	82,2	88,4	56,8	86,9	84,0
Raça/cor									
	Não negra	77,8	61,3	84,4	89,3	79,8	39,3	86,8	82,1
	Negra	74,6	56,5	82,5	81,9	79,1	39,0	84,6	77,7
Escolaridade									
	Ensino fundamental	76,1	61,8	74,3	80,7	79,0	40,5	79,3	75,5
	Ensino médio completo e incompleto	75,1	57,4	84,1	84,9	80,0	37,7	84,9	80,3
	Ensino superior e mais	73,7	54,3	86,8	87,8	77,9	39,9	89,0	81,6
Trabalho remunerado									
	Sim	71,8	54,6	83,4	85,0	77,9	38,4	85,8	79,8
	Não	80,6	67,5	81,5	82,3	81,6	40,8	83,1	77,2
Número de cômodos por morador									
	< 2	74,6	57,8	80,5	83,6	78,4	36,8	82,6	77,9
	≥ 2	75,2	58,6	84,8	85,4	80,0	42,8	86,9	81,2
Acesso aos serviços de saúde									
	Exclusivamente SUS	74,2	59,0	81,1	84,5	78,8	38,6	83,0	78,9
	Serviço privado, plano de saúde ou todos os serviços	76,4	55,2	86,0	83,5	80,0	40,5	88,5	79,9
Consulta médica									
	Não	73,8	58,0	81,5	81,4	74,3	36,0	83,4	78,9
	Sim	75,2	58,1	83,1	85,1	80,5	40,0	85,3	79,2
Autoavaliação da saúde									
	Ruim/muito ruim	72,9	52,8	80,7	82,2	73,8	36,3	81,9	80,5
	Regular	75,2	60,5	82,7	84,4	80,0	39,2	84,6	80,7
	Muito boa/boa	75,2	58,0	83,6	85,3	80,1	40,6	86,5	76,1
Percepção da chance de ter Covid-19									
	Nenhuma/baixa	80,1	60,7	80,0	82,9	82,3	36,4	84,6	77,6
	Moderada/alta	73,3	56,2	83,6	85,3	78,2	41,0	84,9	80,2
Contatante de pessoa(s) com Covid-19 ou sintomas gripais									
	Não	76,5	60,8	82,6	82,9	78,0	37,6	84,0	78,0
	Sim	73,6	54,6	82,9	86,0	80,3	41,0	85,7	80,5
Residir com pessoa(s) do grupo em maior vulnerabilidade à Covid-19									
	Não	73,0	56,8	82,1	84,4	77,3	36,6	84,1	78,3
	Sim	78,0	60,5	83,7	84,0	82,2	43,9	86,1	80,7
Uso de medicamento sem comprovação científica									
	Não	74,6	57,7	83,2	84,1	79,5	38,4	84,9	79,1
	Sim	76,9	61,6	79,6	85,9	77,0	44,9	84,9	79,3
Número de morbidades autorreferidas									
	Nenhuma	72,7	55,2	82,5	84,2	76,6	35,0	84,2	78,2
	Uma	79,0	61,0	83,1	84,1	83,0	45,5	85,9	79,5
	Duas ou mais	81,1	70,2	83,1	85,7	89,0	49,4	87,1	85,1
Relato de diagnóstico prévio de Covid-19									
	Nenhuma vez	75,5	61,2	81,6	83,3	79,8	37,5	84,0	78,2
	Uma ou mais	74,0	55,1	84,6	85,2	78,2	40,6	86,3	80,0
Tabagismo atual									
	Fumante	69,0	54,1	77,3	77,2	74,0	31,6	78,5	70,9
	Não fumante	75,3	58,9	83,1	85,6	79,5	40,5	85,3	80,7
Vacina influenza									
	Não	71,4	53,1	80,8	81,5	73,1	27,9	80,8	76,4
	Sim	76,6	60,4	83,6	85,6	82,1	44,3	86,8	80,4
Vacina Covid-19									
	Até 2 doses	71,1	49,8	77,1	77,0	72,1	30,2	78,8	70,9
	1º reforço	72,9	55,8	83,0	87,5	76,4	31,4	84,5	79,8
	2º reforço	78,7	63,4	85,3	86,0	85,5	47,6	88,3	82,7

P (%): prevalência em percentual; SUS: Sistema Único de Saúde.

Em Salvador, o distanciamento social foi mais frequente entre participantes de 40 a 59 anos (77,7%) e ≥ 60 anos (84,7%), sem trabalho remunerado (80,6%), que conviviam com pessoas vulneráveis à Covid-19 (78%) ou vacinados contra influenza (76,6%). A percepção moderada/alta de chance de infecção por Covid-19 esteve associada a menor adesão (73,3%). A etiqueta respiratória foi mais prevalente entre mulheres (84%), pessoas de 40–59 anos (84,5%), com ensino médio (84,1%) ou superior (86,8%), com maior densidade domiciliar (84,8%), percepção elevada de risco (83,6%) e que receberam reforço vacinal contra Covid-19 (83% para o 1º reforço e 85,3% para o 2º reforço). Foi menor entre usuários de medicamentos sem comprovação científica (79,6%) (Tabela 2).

O uso de máscara foi mais comum entre mulheres (81,6%), pessoas com 40–59 anos (84,6%) e ≥ 60 anos (88,4%), com autoavaliação de saúde regular (80%) ou boa/muito boa (80,1%), percepção baixa de risco (82,3%), que tiveram contato com casos de Covid-19 (80,3%), conviviam com pessoas vulneráveis (82,2%), relataram morbidades (83,1%), usaram vacina contra influenza (82,1%) e receberam reforço contra Covid-19 (76,4 e 85,5%). A higienização das mãos foi mais frequente entre mulheres (86,4%), pessoas de 40–59 anos (87,5%) e ≥ 60 anos (86,9%), com ensino médio (84,9%) ou superior (89%), que trabalhavam (85,8%), com maior densidade domiciliar (86,9%), melhor autoavaliação de saúde (86,5%), que conviviam com pessoas vulneráveis (86,1%) e se vacinaram contra influenza (86,8%) e Covid-19 (84,5 e 88,3%) (Tabela 2).

No Rio de Janeiro, a prevalência da adoção do distanciamento social foi maior entre os participantes com idade de 40–59 anos (61,8%) e ≥ 60 anos (68,3%), que não trabalhavam (67,5%), com autoavaliação regular da saúde (60,5%), que não tiveram contato com casos de Covid-19 (60,8%), sem relato prévio de diagnóstico de Covid-19 (61,2%) e que receberam o reforço contra Covid-19 (1º reforço = 55,8%; 2º reforço = 63,4%). A etiqueta respiratória foi mais frequente entre as mulheres (86,4%), pessoas não negras (89,3%), com ensino médio (84,9%) ou superior e mais (87,8%), não tabagistas (85,6%), e que receberam reforço contra Covid-19 (1º reforço = 87,5%; 2º reforço = 86,0%). O uso de máscara foi mais prevalente entre as mulheres (42,3%), com maior idade (40–59 anos = 41,9%; ≥ 60 anos = 56,8%), percepção moderada/alta de chance de infecção por Covid-19 (41%), que utilizaram a vacina contra influenza (44,3%) e receberam reforço contra Covid-19 (1º reforço = 31,4%; 2º reforço = 47,6%). A higienização das mãos foi maior entre as mulheres (81,6%), com idade de 40 a 59 (82,3%) e ≥ 60 anos (84,0%), com ensino médio (80,3%) ou superior e mais (81,6%), que receberam reforço vacinal contra Covid-19 (1º reforço = 79,8%; 2º reforço = 82,7%) (Tabela 2).

Após análise múltipla, no município de Salvador, o sexo feminino, a maior idade e escolaridade, a percepção da chance de ter infecção por Covid-19 moderada/alta e o reforço vacinal contra Covid-19 permaneceram significativamente associados à adoção da maioria dos comportamentos avaliados (Tabela 3). No Rio de Janeiro, por sua vez, o sexo feminino, a maior idade e o reforço vacinal contra Covid-19 foram associados a maior prevalência de adoção da maior parte dos comportamentos preventivos (Tabela 4).

**Tabela 3:** Análise bivariada e múltipla entre as variáveis explicativas e os comportamentos preventivos à Covid-19 em Salvador/BA (n = 5.476). Estudo TQT Covid-19, Brasil, 2023.

Variáveis		Salvador (BA)							
		Distanciamento social		Etiqueta respiratória		Barreira por máscara		Higienização das mãos	
		RP (IC95%)	RP_a_ (IC95%)	RP (IC95%)	RP_a_ (IC95%)	RP (IC95%)	RP_a_ (IC95%)	RP (IC95%)	RP_a_ (IC95%)
Sexo									
	Masculino	1		1	1	1	1	1	1
	Feminino	**1,04** (1,01–1,07)		**1,05** (1,02–1,08)	**1,05** (1,02–1,08)	**1,11** (1,08–1,15)	**1,09** (1,06–1,14)	**1,06** (1,04–1,09)	**1,06** (1,03–1,09)
Idade (anos)									
	18–39	1	1	1	1	1	1	1	1
	40–59	**1,12** (1,08–1,16)	**1,12** (1,08–1,16)	**1,04** (1,01–1,07)	**1,04** (1,01–1,07)	**1,18** (1,14–1,21)	**1,14** (1,11–1,18)	**1,07** (1,04–1,09)	**1,06** (1,03–1,09)
	≥ 60	**1,22** (1,17–1,27)	**1,16** (1,11–1,21)	1,01 (0,97–1,05)	1,03 (0,99–1,07)	**1,23** (1,19–1,27)	**1,15** (1,10–1,20)	**1,06** (1,02–1,09)	**1,06** (102–1,11)
Raça/cor									
	Não negra	1		1		1		1	
	Negra	0,96 (0,91–1,01)		0,98 (0,94–1,01)		0,99 (0,95–1,04)		0,98 (0,94–1,01)	
Escolaridade									
	Ensino fundamental	1	1	1	1	1		1	1
	Ensino médio completo e incompleto	0,99 (0,95–1,02)	**1,04** (1,01–1,08)	**1,13** (1,09–1,17)	**1,14** (1,09–1,18)	1,01 (0,98–1,05)		**1,07** (1,04–1,11)	**1,08** (1,04–1,11)
	Ensino superior e mais	0,97 (0,93–1,01)	1,04 (0,99–1,09)	**1,17** (1,12–1,21)	**1,16** (1,11–1,21)	0,99 (0,95–1,03)		**1,12** (1,08–1,16)	**1,11** (1,07–1,15)
Trabalho remunerado									
	Sim	1	1	1		1		1	1
	Não	**1,12** (1,09–1,16)	**1,09** (1,05–1,12)	0,98 (0,95–1,01)		**1,05** (1,02–1,08)		**0,98** (0,94–0,99)	**0,96** (0,94–0,99)
Número de cômodos por morador									
	< 2	1		1	1	1		1	1
	≥ 2	1,01 (0,98–1,04)		**1,05** (1,03–1,08)	**1,03** (1,01–1,06)	1,02 (0,99–1,05)		**1,05** (1,03–1,08)	**1,04** (1,01–1,06)
Acesso aos serviços de saúde									
	Exclusivamente SUS	1		1		1		1	
	Serviço privado, plano de saúde ou todos os serviços	1,03 (0,99–1,06)		**1,06** (1,03–1,09)		1,01 (0,99–1,04)		**1,06** (1,04–1,09)	
Consulta médica									
	Não	1		1		1		1	
	Sim	1,02 (0,98–1,06)		1,02 (0,99–1,05)		**1,08** (1,04–1,12)		1,02 (0,99–1,05)	
Autoavaliação da saúde									
	Ruim/muito ruim	1		1		1	1	1	1
	Regular	1,03 (0,98–1,08)		1,02 (0,98–1,06)		**1,08** (1,04–1,13)	**1,07** (1,02–1,12)	1,03 (0,99–1,07)	1,03 (0,99–1,07)
	Muito boa/boa	1,03 (0,98–1,09)		1,04 (0,99–1,09)		**1,09** (1,03–1,14)	**1,08** (1,03–1,13)	**1,06** (1,01–1,10)	**1,04** (1,01–1,08)
Percepção da chance de ter Covid-19									
	Nenhuma/baixa	1	1	1	1	1	1	1	
	Moderada/alta	**0,91** (0,88–0,94)	**0,93** (0,90–0,96)	**1,04** (1,01–1,08)	**1,03** (1,01–1,06)	**0,95** (0,92–0,98)	**0,96** (0,93–0,99)	1,01 (0,98–1,03)	
Contatante de pessoa(s) com Covid-19 ou sintomas gripais									
	Não	1		1		1	1	1	
	Sim	**0,96** (0,93–0,99)		1,01 (0,98–1,03)		**1,03** (1,01–1,06)	**1,03** (1,01–1,06)	1,02 (0,99–1,04)	
Residir com pessoa(s) do grupo em maior vulnerabilidade à Covid-19									
	Não	1	1	1		1	1	1	1
	Sim	**1,07** (1,03–1,10)	**1,06** (1,03–1,10)	1,02 (0,99–1,04)		**1,06** (1,03–1,09)	**1,06** (1,03–1,10)	**1,02** (1,01–1,05)	**1,03** (1,01–1,05)
Uso de medicamento sem comprovação científica									
	Não	1		1	1	1		1	
	Sim	1,03 (0,98–1,08)		**0,96** (0,92–0,99)	**0,95** (0,92–0,99)	0,97 (0,93–1,01)		1,01 (0,97–1,03)	
Número de morbidades autorreferidas									
	Nenhuma	1		1		1	1	1	
	Uma	**1,09** (1,05–1,12)		1,01 (0,99–1,04)		**1,08** (1,05–1,12)	1,01 (0,98–1,04)	1,02 (0,99–1,05)	
	Duas ou mais	**1,11** (1,06–1,17)		1,01 (0,97–1,05)		**1,16** (1,12–1,20)	**1,05** (1,01–1,10)	1,03 (0,99–1,07)	
Relato de diagnóstico prévio de Covid-19									
	Nenhuma vez	1		1		1		1	
	Uma ou mais	0,98 (0,95–1,01)		**1,04** (1,01–1,06)		0,98 (0,95–1,01)		**1,03** (1,01–1,05)	
Tabagismo atual									
	Fumante	1		1		1		1	
	Não fumante	**1,09** (1,01–1,17)		**1,07** (1,01–1,14)		**1,07** (1,01–1,15)		**1,09** (1,03–1,15)	
Vacina influenza									
	Não	1	1	1		1	1	1	1
	Sim	**1,07** (1,04–1,11)	**1,04** (1,01–1,08)	**1,04** (1,01–1,06)		**1,12** (1,09–1,16)	**1,06** (1,03–1,10)	**1,07** (1,05–1,10)	**1,05** (1,02–1,07)
Vacina Covid-19									
	Até 2 doses	1		1	1	1	1	1	1
	1º reforço	1,02 (0,98–1,07)		**1,07** (1,04–1,12)	**1,05** (1,01–1,09)	**1,06** (1,01–1,11)	1,03 (0,98–1,07)	**1,07** (1,03–1,11)	**1,04** (1,01–1,08)
	2º reforço	**1,11** (1,06–1,15)		**1,11** (1,07–1,15)	**1,07** (1,04–1,12)	**1,18** (1,14–1,23)	**1,08** (1,03–1,13)	**1,12** (1,08–1,16)	**1,06** (1,02–1,10)

RP: razão de prevalência; RP_a_: razão de prevalência ajustada; IC95%: intervalo de confiança de 95%.

Nota: em negrito, resultados significativos com p < 0,05.

**Tabela 4:** Análise bivariada e múltipla entre as variáveis explicativas e os comportamentos preventivos à Covid-19 no Rio de Janeiro (RJ) (n = 1.940). Estudo TQT Covid-19, Brasil, 2023.

Variáveis		Rio de Janeiro (RJ)							
		Distanciamento social		Etiqueta respiratória		Barreira por máscara		Higienização das mãos	
		RP (IC95%)	RP_a_ (IC95%)	RP (IC95%)	RP_a_ (IC95%)	RP (IC95%)	RP_a_ (IC95%)	RP (IC95%)	RP_a_ (IC95%)
Sexo									
	Masculino	1		1	1	1	1	1	1
	Feminino	1,08 (0,99–1,17)		**1,08** (1,03–1,13)	**1,07** (1,02–1,12)	**1,30** (1,15–1,48)	**1,23** (1,08–1,40)	**1,10** (1,04–1,16)	**1,09** (1,03–1,15)
Idade (anos)									
	18–39	1	1	1		1	1	1	1
	40–59	**1,20** (1,10–1,31)	**1,19** (1,09–1,30)	1,01 (0,97–1,05)		**1,39** (1,22–1,58)	**1,29** (1,12–1,47)	**1,10** (1,04–1,16)	**1,10** (1,04–1,16)
	≥ 60	**1,33** (1,21–1,46)	**1,18** (1,06–1,31)	0,97 (0,92–1,03)		**1,88** (1,64–2,16)	**1,64** (1,42–1,89)	**1,12** (1,06–1,19)	**1,13** (1,06–1,21)
Raça/cor									
	Não negra	1		1	1	1		1	
	Negra	**0,92** (0,85–0,99)		**0,92** (0,88–0,95)	**0,93** (0,89–0,96)	0,99 (0,88–1,12)		**0,95** (0,90–0,99)	
Escolaridade									
	Ensino fundamental	1		1	1	1		1	1
	Ensino médio completo e incompleto	0,93 (0,85–1,01)		**1,05** (1,01–1,10)	**1,05** (1,01–1,10)	0,93 (0,82–1,06)		**1,06** (1,01–1,13)	**1,10** (1,04–1,17)
	Ensino superior e mais	**0,88** (0,79–0,98)		**1,09** (1,03–1,15)	1,05 (0,99–1,11)	0,98 (0,84–1,15)		**1,08** (1,01–1,15)	**1,10** (1,04–1,19)
Trabalho remunerado									
	Sim	1	1	1		1		1	
	Não	**1,24** (1,15–1,34)	**1,17** (1,08–1,27)	0,97 (0,93–1,01)		1,06 (0,94–1,20)		0,97 (0,92–1,02)	
Número de cômodos por morador									
	< 2	1		1		1		1	
	≥ 2	1,01 (0,94–1,09)		1,02 (0,98–1,06)		**1,16** (1,04–1,30)		1,04 (0,99–1,09)	
Acesso aos serviços de saúde									
	Exclusivamente SUS	1		1		1		1	
	Serviço privado, plano de saúde ou todos os serviços	0,93 (0,85–1,03)		0,99 (0,94–1,03)		1,05 (0,92–1,20)		1,01 (0,96–1,07)	
Consulta Médica									
	Não	1		1		1		1	
	Sim	1,01 (0,91–1,10)		1,04 (0,99–1,10)		1,11 (0,96–1,28)		1,01 (0,95–1,06)	
Autoavaliação da saúde									
	Ruim/muito ruim	1	1	1		1		1	
	Regular	**1,14** (1,03–1,27)	**1,14** (1,03–1,27)	1,03 (0,97–1,08)		1,08 (0,93–1,26)		1,01 (0,95–1,06)	
	Muito boa/boa	1,10 (0,98–1,23)	1,10 (0,99–1,23)	1,04 (0,98–1,10)		1,12 (0,95–1,31)		0,95 (0,89–1,01)	
Percepção da chance de ter Covid-19									
	Nenhuma/baixa	1		1		1	1	1	
	Moderada/alta	**0,93** (0,86–0,99)		1,03 (0,99–1,07)		**1,12** (1,01–1,26)	**1,13** (1,01–1,27)	1,03 (0,99–1,08)	
Contatante de pessoa(s) com Covid-19 ou sintomas gripais									
	Não	1	1	1		1		1	
	Sim	**0,90** (0,83–0,97)	**0,92** (0,85–0,99)	**1,04** (1,01–1,08)		1,09 (0,97–1,22)		1,03 (0,99–1,08)	
Residir com pessoa(s) do grupo em maior vulnerabilidade à Covid-19									
	Não	1		1		1		1	
	Sim	1,06 (0,98–1,15)		0,99 (0,95–1,04)		**1,20** (1,07–1,34)		1,03 (0,98–1,08)	
Uso de medicamento sem comprovação científica									
	Não	1		1		1		1	
	Sim	1,07 (0,95–1,20)		1,02 (0,96–1,08)		1,17 (0,99–1,38)		1,01 (0,93–1,08)	
Número de morbidades autorreferidas									
	Nenhuma	1		1		1		1	
	Uma	**1,10** (1,01–1,20)		0,99 (0,96–1,04)		**1,30** (1,15–1,47)		1,01 (0,96–1,07)	
	Duas ou mais	**1,27** (1,14–1,42)		1,02 (0,95–1,09)		**1,41** (1,19–1,67)		**1,09** (1,01–1,17)	
Relato de diagnóstico prévio de Covid-19									
	Nenhuma vez	1	1	1		1		1	
	Uma ou mais	**0,90** (0,83–0,97)	**0,90** (0,84–0,98)	1,02 (0,98–1,06)		1,08 (0,97–1,21)		1,02 (0,98–1,07)	
Tabagismo atual									
	Fumante	1		1	1	1		1	
	Não fumante	1,09 (0,97–1,21)		**1,11** (1,04–1,18)	**1,08** (1,02–1,15)	**1,28** (1,08–1,52)		**1,14** (1,06–1,23)	
Vacina influenza									
	Não	1		1		1	1	1	
	Sim	**1,14** (1,04–1,24)		**1,05** (1,01–1,10)		**1,59** (1,38–1,83)	**1,32** (1,14–1,53)	1,05 (0,99–1,11)	
Vacina Covid-19									
	Até 2 doses	1	1	1	1	1	1	1	1
	1º reforço	1,12 (0,99–1,26)	**1,13** (1,01–1,27)	**1,14** (1,07–1,21)	**1,12** (1,06–1,19)	1,04 (0,86–1,25)	0,97 (0,81–1,17)	**1,13** (1,05–1,21)	**1,11** (1,04–1,19)
	2º reforço	**1,27** (1,15–1,41)	**1,21** (1,09–1,35)	**1,12** (1,06–1,18)	**1,10** (1,04–1,16)	**1,58** (1,35–1,84)	**1,21** (1,03–1,43)	**1,17** (1,09–1,25)	**1,12** (1,04–1,19)

RP: razão de prevalência; RP_a_: razão de prevalência ajustada; IC 95%: intervalo de confiança de 95%.

Nota: em negrito, resultados significativos com p < 0,05.

## DISCUSSÃO

Este estudo identificou que a etiqueta respiratória e a higienização das mãos foram os comportamentos preventivos com maiores prevalências de adoção no período regular da pandemia, tanto em Salvador quanto no Rio de Janeiro. O sexo feminino, a maior idade e escolaridade, a utilização das vacinas oferecidas ou as suas doses de reforço foram as variáveis que mais aumentaram as prevalências dos comportamentos preventivos avaliados, que sofreram mudanças após o pico pandêmico.

Nossos achados evidenciaram que o uso de máscara facial e o distanciamento físico obtiveram menor adoção no período regular da pandemia, principalmente no Rio de Janeiro. Outro estudo realizado também em Manguinhos, no pico pandêmico, identificou baixa adesão ao distanciamento social e elevada adoção do uso de máscara^13^. O estudo EpiCovid-19 Brasil, de 2020, revelou altas prevalências de distanciamento social e uso de máscaras ao sair de casa, tanto por portadores de doenças crônicas não transmissíveis quanto por não portadores^9^. Destaca-se que a coleta de dados ocorreu em períodos distintos em Salvador e no Rio de Janeiro. Nesta última, ocorreu durante maior controle da Covid-19, com vacinação ampla, e continuou após o término oficial da pandemia. Os diferentes períodos podem explicar essas variações nas prevalências dos comportamentos, além de diferenças metodológicas empregadas.

As participantes do sexo feminino adotaram mais comportamentos preventivos à Covid-19 neste estudo, de forma consistente com estudos em outros locais^14-18^. Uma metanálise revelou que as mulheres têm cerca de 50% mais chances do que os homens de adotar/praticar medidas não farmacológicas^14^. Os cuidados durante a pandemia de Covid-19 foram desiguais por gênero, sendo as mulheres mais propensas a encarar a pandemia como um problema de saúde grave e a cumprir medidas de restrição^15^. No Chile, elas relataram os mais altos níveis de preocupação e medo sobre a possibilidade de infecção e propagação do vírus, adotando mais medidas preventivas^16^. Uma pesquisa com dados de 175 países mostrou que as mulheres apresentaram maior adesão aos comportamentos de proteção da saúde contra a Covid-19^17^. Esses achados reforçam a necessidade de levar em consideração o gênero nas campanhas de comunicação de risco.

A maior idade favoreceu a adoção dos diversos comportamentos avaliados, exceto para etiqueta respiratória no Rio de Janeiro. Estudos mostraram que a infecção por Covid-19 tem maior gravidade e maior taxa de mortalidade entre os indivíduos de maior idade^19^, especialmente entre os idosos, o que contribui para maior ameaça percebida por essa população. Esse achado é corroborado por outros trabalhos que apontam que os indivíduos mais velhos apresentam maior percepção de risco em relação à Covid-19^20^ e maior probabilidade de adoção de comportamentos protetores^21^.

Maior nível de escolaridade foi um fator associado ao aumento da prevalência na adoção da etiqueta respiratória e higienização das mãos, o que tinha sido observado em outros estudos^20-23^. Na África do Sul, a maior percepção de risco em relação à pandemia da Covid-19 estava presente entre os indivíduos com oito ou mais anos de estudo e entre aqueles grupos com maiores rendas^20^. Ozdemir et al.^21^ mostraram que a maior escolaridade aumentou a frequência da adoção de diversos comportamentos preventivos. Fang et al.^22^ relacionaram a escolaridade ao impacto positivo na adoção de comportamentos preventivos no pico pandêmico, entretanto, diferindo dos nossos achados, não verificaram esse efeito em épocas de epidemia regular.

Não ter exercido trabalho remunerado nos últimos 12 meses foi associado a maior prevalência do distanciamento social em ambos os sítios. Szwarcwald et al.^7^ relataram que trabalhadores têm maior dificuldade em manter o distanciamento social, e isso pode repercutir na redução da adoção de alguns comportamentos preventivos. Em uma análise com minorias sexuais e de gênero do Brasil nos primeiros meses da pandemia, observou-se uma relação entre a maior vulnerabilidade e a dificuldade de manter distanciamento social, provavelmente por precisarem sair para trabalhar, visto que havia menos opção de home office para esses grupos^23^.

Dentre as variáveis de suscetibilidade da Covid-19, a percepção moderada/alta da chance de infecção teve efeito aumentando ou diminuindo alguns dos comportamentos preventivos. É possível que essa variável tenha uma relação causal bidirecional com a adesão, visto que, em função de não conseguir aderir a um determinado comportamento, o indivíduo percebe-se com maior chance de adoecer. Isso explicaria o menor distanciamento social e a utilização de barreira por máscara facial em Salvador. Ressalta-se que, no período regular da pandemia, algumas medidas preventivas haviam sido flexibilizadas, em função dos benefícios advindos do aumento da cobertura vacinal^24^. Durante a coleta de dados no Rio de Janeiro, observou-se também um sistema de saúde público mais consolidado e organizado para atender à população.

Em face a flexibilização das medidas e menor incidência da Covid-19, essa doença não deixou de ser uma ameaça à saúde^24^. O medo ainda existente à exposição ao vírus pode ter aumentado a adesão aos comportamentos de distanciamento social, barreira por máscara e higienização das mãos em Salvador pelos participantes que residiam com pessoa em situação de vulnerabilidade para a doença. Silva et al.^25^, no estado do Ceará, verificaram a maior adesão ao distanciamento social entre os participantes que sentiam medo da exposição dos familiares ao risco da infecção.

O uso de medicamento sem comprovação científica para tratar ou prevenir a Covid-19 teve baixa prevalência em nosso estudo, entretanto essa prática foi associada negativamente à adoção da etiqueta respiratória em Salvador. É importante destacar que este estudo foi realizado no período marcado por mudanças políticas no Brasil e na fase regular da pandemia, de menor mortalidade e incidência de novos casos de contaminação pelo vírus. Na gestão anterior do governo brasileiro, o Ministério da Saúde enfrentou fortes limitações financeiras e, no período pandêmico, o sistema público de saúde encontrava-se desarticulado, desestruturado e enfrentava um processo de desmonte^26^. Houve desestímulo às medidas preventivas e incitação às medidas sem comprovação científica, como o uso de medicamentos para tratar ou prevenir a Covid-19.

No primeiro ano do atual governo (2023), as estratégias na área da saúde para a reestruturação do Ministério da Saúde visaram fortalecer o corpo técnico e científico da gestão e coordenação do SUS, recuperar o Programa Nacional de Imunização e intensificar a resposta à Covid-19^27^. Uma das estratégias foi garantir a continuidade da imunização da população contra a Covid-19 e a influenza, observada na maioria dos participantes em nosso estudo, em ambos os sítios, com alta adesão à dose de reforço do imunizante contra a Covid-19. A utilização dessas vacinas mostrou-se associada a maior adoção dos diversos comportamentos avaliados em Salvador e no Rio de Janeiro.

Este estudo tem algumas limitações. Os dados foram autorreferidos, sujeitos então a vieses, como de informação, memória e de desejabilidade social, podendo sofrer influência de comportamentos socialmente aceitáveis e superestimar a adoção de alguns dos comportamentos em saúde. Comparando as amostras dos dois municípios com a população obtida no último censo demográfico, observamos diferenças em relação a sexo, idade e raça, com maior predominância de mulheres, pessoas negras e mediana da idade superior à população geral^28^. Como este estudo teve amostra por conveniência, com participação voluntária de acordo com unidades de saúde, essas diferenças já eram esperadas. Assim, o processo de amostragem empregado pode ter levado a uma amostra não representativa da população das regiões avaliadas, visto que apenas aqueles com efetivo acesso aos serviços foram incluídos. Entretanto, as estratégias utilizadas para sensibilizar a população-alvo foram bem diversas, o que aumentou a probabilidade de inclusão dos diversos estratos sociais.

Como pontos fortes, destacam-se a realização desta pesquisa no período regular da pandemia e em populações socialmente vulneráveis, diferindo da maioria das pesquisas acerca dos comportamentos preventivos à Covid-19, possibilitando identificar os comportamentos que foram mantidos após o pico pandêmico.

Nossos achados contribuem significativamente para a saúde pública ao identificar barreiras e facilitadores na adesão de comportamentos preventivos à Covid-19, fornecendo subsídios para reduzir desigualdades no acesso à saúde. Além disso, oferecem análises valiosas para sensibilizar a sociedade quanto à prevenção de futuras epidemias, fortalecendo a capacidade de resposta em populações de maior vulnerabilidade. Essas ações também devem mobilizar as redes de atenção à saúde para uma educação comunitária contínua, visando capacitar a população, combater a desinformação e fomentar uma cultura de prevenção e cuidado em saúde pública.

Em conclusão, este estudo identificou alta adesão dos comportamentos preventivos no período regular da pandemia. Observou-se a manutenção dos comportamentos, com adesão acima do esperado, especialmente da etiqueta respiratória e higienização das mãos, enquanto o uso de máscara e o distanciamento social reduziram como previsto, mais pronunciadamente no Rio de Janeiro. As mulheres, a maior idade, maior escolaridade e a adesão vacinal aumentaram a adoção de comportamentos preventivos à Covid-19.

## Data Availability

Os conjuntos de dados gerados e/ou analisados neste estudo não estão disponíveis publicamente por integrarem um projeto mais amplo, com outras análises em desenvolvimento. Contudo, podem ser obtidos com o autor correspondente mediante solicitação razoável.
